# You are what you eat: feeding neurons in nutrient regulation of behavior

**DOI:** 10.1093/lifemeta/load006

**Published:** 2023-02-08

**Authors:** Jessie C Morrill, Qingchun Tong

**Affiliations:** Department of Animal Science, University of Nebraska, Lincoln, Lincoln, NE 68503, USA; Brown Foundation Institute of Molecular Medicine for the Prevention of Human Diseases, McGovern Medical School of the University of Texas Health Science Center at Houston, TX 77030, USA

**In a recent paper published in *Life Metabolism*, Yuan *et al.* demonstrated that deprivation of the essential amino acid, leucine, alleviated depressive behaviors that were induced by chronic stress. Specifically, the antidepressant effects were shown to be mediated by the activation of agouti-related protein (AgRP)-expressing neurons, which are known for the ability to sense bodily energy status and promote energy intake, revealing a neural basis for the availability of nutrients in controlling mental behaviors.**

The brain has evolved to be capable of efficiently adapting to dynamic changes in the environment and internal bodily needs and adjusts behaviors to increase odds of survival. Research from last few decades has identified the critical roles of hypothalamic neurons, including agouti-related protein (AgRP) neurons, in sensing bodily nutritional status, and in turn, adjusting feeding behaviors to maintain body weight homeostasis. Specifically, AgRP neurons are capable of directly sensing a variety of nutritional status indicators including leptin, glucose, insulin, and ghrelin, and subsequently change firing activity to modulate food intake and body weight [1]. More recent studies suggest that the conventional food intake-regulating neurons are also involved in modulating behavioral signs of mental states including anxiety and depression. Specifically, activation of AgRP neurons reduces behavioral signs of anxiety and increases motivation and explorative behaviors [2–4]. These observations suggest that a common neural pathway coordinates the responses to both internal nutritional status and external cues to generate a concerted behavioral repertoire for better survival. These observations also underscore a neural basis for the strong association of anorexia with anxiety and of obesity with altered mental states [5]. However, in contrast to the well-established regulation of AgRP neurons by nutritional signals, the sensing mechanisms for these neurons in modulating mental behaviors are less understood. The study by Yuan *et al.* [6] provides strong evidence supporting essential animal acid (EAA) sensing as one of the mechanisms underlying hypothalamic AgRP neurons in adapting nutrient intake to control mental behaviors.

The authors first established an animal model with depressive behavioral signs of anxiety and depression through a 21-day chronic restraint stress (CRS) protocol. Interestingly, a short subsequent 3-day period feeding with diets that were formulated to be deficient in each of the EAAs examined was shown to reduce all CRS-induced behavioral signs of depression. This reduction can be reversed by subsequent intake of normal diet with EAAs, suggesting a causal role for EAA deficiency on the effect. To examine the underlying mechanism, the authors then focused on leucine (Leu), a branched chain EAA. Since Leu deficiency was also associated with reduced feeding, additional pair-feeding studies ruled out the potential contribution from reduced feeding, suggesting a specific nutrient-sensing mechanism involved in the observed behavioral adaptation. EAA sensing can occur in both peripheral sites and the brain. To differentiate the effect in the brain from the periphery, the authors performed intracerebroventricular injection of Leu to specifically target the brain. Brain injection of Leu effectively abrogated the beneficial effect of dietary Leu deficiency on depressive behaviors, pointing to a direct effect of brain neurons. This point was further substantiated by a similar depressive behavioral phenotype induced by brain injection of Leucinol, an agent that mimics dietary Leu deficiency.

Due to the known implications of hypothalamic AgRP neurons in both nutrient-sensing and depressive behaviors [2, 4], additional studies were then conducted to investigate the role of AgRP neurons in the observed depressive behaviors by availability of dietary Leu. Immunostaining results demonstrated that the CRS protocol caused a significant reduction in the expression of several early genes, including c-Fos, a known marker of neuron activation, which was reversed by Leu deprivation. Importantly, the changes in neural activity were most notably found in AgRP neurons. This finding substantiates AgRP neurons as a potential key player in mediating the behavioral effect by dietary Leu deficiency. In additional experiments, chemogenetic manipulation of AgRP neurons further demonstrated that specific activation of AgRP neurons alleviated depressive behaviors, mimicking Leu deficiency, whereas specific inhibition reversed the effect, suggesting that AgRP neuron activation is a major mechanism involved in mediating the effect of Leu deficiency.

It is intriguing that Leu deprivation-induced AgRP neuronal activation failed to increase feeding, which is somewhat inconsistent with the well-established notion that activated AgRP neurons promote feeding [7]. This discrepancy may be caused by an altered depressive state induced by CRS. Alternatively, since AgRP neurons send parallel and largely non-overlapping projections to a number of different downstream brain sites [8], it is possible that a subset of AgRP neurons become activated in dietary Leu deprivation, which are distinct from the subset that promotes feeding.

EAAs can be sensed by brain neurons through a variety of signaling mechanisms. The most well-studied signaling mechanisms include the general control non-derepressible 2 (GCN2) and the mammalian target of rapamycin (mTOR) pathways, both of which are known to regulate protein translation in response to the availability of amino acids [9]. In this study, the authors primarily focused on the GCN2 pathway with a specific knockdown approach. They demonstrated that GCN2 in AgRP neurons was required in alleviating depressive behaviors by Leu deprivation which was associated with reduced activation of AgRP neurons, strongly supporting a major role of GCN2 in sensing Leu deficiency in AgRP neuron activation. Given the known role of the mTOR pathway in sensing amino acid abundance, it is worth contemplating a potential involvement of mTOR in the study paradigm that was presented. On this point, Leu has been previously shown to reduce feeding and body weight through activation of the mTOR pathway in the hypothalamus [10].Future studies are warranted to examine how these distinct nutrient-sensing pathways are coordinated to affect neuron activity and animal behavior.

**Figure 1:**
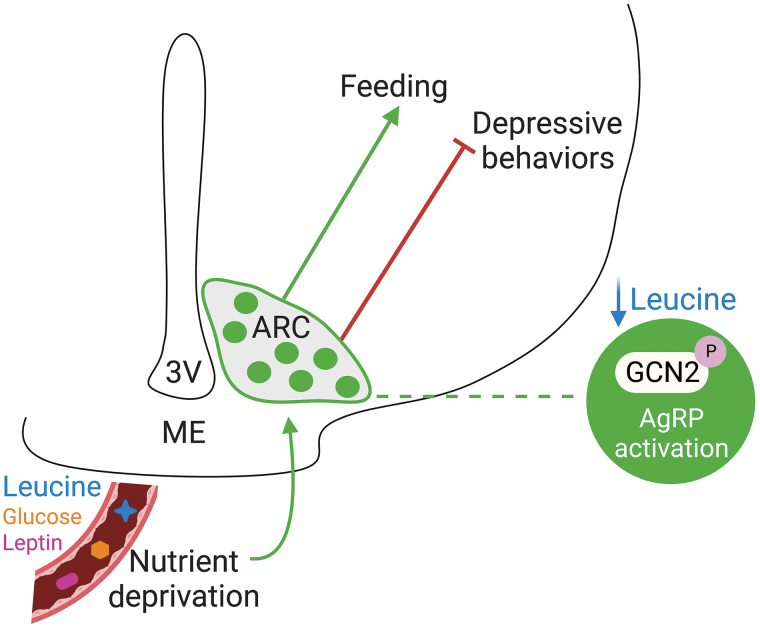
Agouti-related protein (AgRP)-expressing neurons within the arcuate (ARC) hypothalamus sense nutrient availability; low circulating levels of essential amino acids (EAAS), including leucine, and low levels of glucose and leptin result in activation of AgRP neurons and subsequent promotion of feeding and reduction of depressive behaviors. Deprivation of leucine reduces depressive behaviors via a general control non-derepressible 2 (GCN2)-mediated mechanism in AgRP neurons. Illustration is created using Biorender.

In summary, Yuan *et al.* provided a comprehensive set of data and convincingly demonstrated that dietary EAA deficiency alleviates CRS-induced depressive behavioral symptoms through activation of AgRP neurons (Fig. 1). Activation of AgRP neurons may represent a common mechanism to promote survival during harsh conditions including fasting. It is conceivable that, during fasting, reduced availability of nutrition activates AgRP neurons to promote feeding as a means of replenishing energy; and reduced availability of EAA detected by AgRP neurons results in overcoming anxiety and fear that may otherwise have prevented food-seeking behavior, as an increase in anxiety and fear normally accompanies food-seeking behavior in wild. Intermittent fasting is shown to benefit health, increase longevity, and reduce depressive symptoms [11].Since the EAA deficiency used in this study is acute, coinciding with the intermittent fasting condition, the current results may support potential importance of EAA deficiency to the benefit of intermittent fasting.
